# Phonon-Mediated and Weakly Size-Dependent Electron and Hole Cooling in CsPbBr_3_ Nanocrystals Revealed by Atomistic Simulations and Ultrafast Spectroscopy

**DOI:** 10.1021/acs.nanolett.9b05051

**Published:** 2020-02-12

**Authors:** Simon C. Boehme, Stephanie ten Brinck, Jorick Maes, Nuri Yazdani, Felipe Zapata, Kai Chen, Vanessa Wood, Justin M. Hodgkiss, Zeger Hens, Pieter Geiregat, Ivan Infante

**Affiliations:** † Department of Theoretical Chemistry, Faculty of Science, Vrije Universiteit Amsterdam, De Boelelaan 1083, 1081 HV Amsterdam, The Netherlands; ‡ Department of Chemistry, Faculty of Sciences, Universiteit Gent, Krijgslaan 281, 9000 Gent, Belgium; ¶ Materials and Device Engineering Group, Department of Information Technology and Electrical Engineering, ETH Zurich, GH 8092 Zurich, Switzerland; ⊥ Netherlands eScience Center, Science Park 140 (Matrix I), 1098 XG Amsterdam, The Netherlands; ∥ The MacDiarmid Institute for Advanced Materials and Nanotechnology, School of Chemical and Physical Sciences, Victoria University of Wellington, 6012 Wellington, New Zealand; ○ Department of Nanochemistry, Istituto Italiano di Tecnologia, Via Morego 30, 16163 Genova, Italy

**Keywords:** Charge-carrier cooling, hot carriers, lead-halide perovskite nanocrystal, electron−phonon coupling, excited-states dynamics, nonadiabatic molecular dynamics

## Abstract

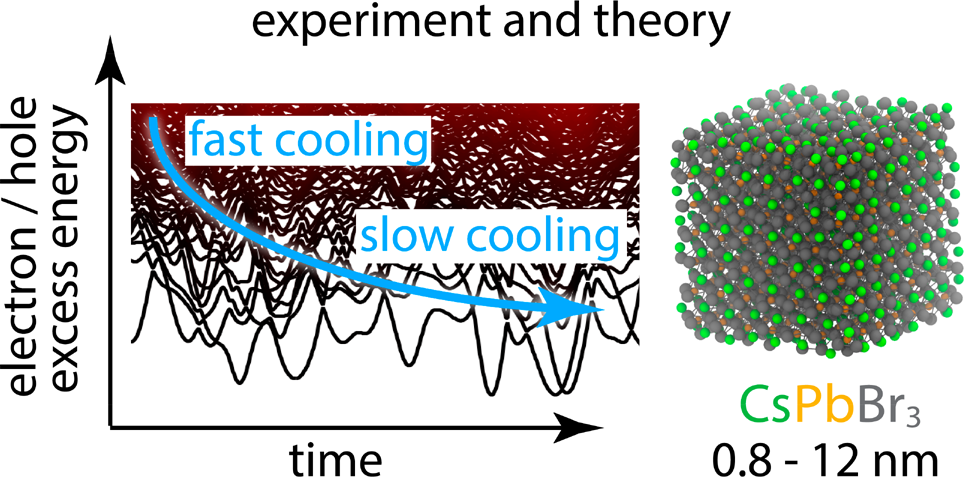

We combine state-of-the-art ultrafast photoluminescence and absorption spectroscopy and nonadiabatic molecular dynamics simulations to investigate charge-carrier cooling in CsPbBr_3_ nanocrystals over a very broad size regime, from 0.8 to 12 nm. Contrary to the prevailing notion that polaron formation slows down charge-carrier cooling in lead-halide perovskites, no suppression of carrier cooling is observed in CsPbBr_3_ nanocrystals except for a slow cooling (over ∼10 ps) of “warm” electrons in the vicinity (within ∼0.1 eV) of the conduction band edge. At higher excess energies, electrons and holes cool with similar rates, on the order of 1 eV ps^–1^ carrier^–1^, increasing weakly with size. Our ab initio simulations suggest that cooling proceeds via fast phonon-mediated intraband transitions driven by strong and size-dependent electron–phonon coupling. The presented experimental and computational methods yield the spectrum of involved phonons and may guide the development of devices utilizing hot charge carriers.

CsPbBr_3_ perovskite nanocrystals (NCs)^1^ have emerged as promising building blocks for optoelectronics, ranging from LEDs, lasers, or solar cells, to single-photon sources, owing to their high defect tolerance, high luminescence quantum yield, narrow luminescence line width, and ease of spectral tuning via (postsynthetic) composition control.^2^ Recent reports pointing out surprisingly long lifetimes of hot charge carriers,^3^ similar to those observed in thin-film lead-halide perovskites (LHPs),^4,5^ furthermore reiterated prospects for a variety of high-efficiency applications relying on hot electrons, e.g., hot-electron catalysis,^6,7^ hot-electron charge extraction,^8,9^ or carrier multiplication.^10,11^ At present, however, a lack of understanding of and control over charge-carrier cooling in LHPs limits the exploitation of such processes in devices.

In bulk and thin-film LHPs, several propositions have been made to explain the observed slow cooling, including arguments based on the phonon density of states (DOS),^12,13^ Auger heating,^3,14,15^ upconversion of acoustic to optical phonons,^16^ surface passivation,^17^ and formation of Fröhlich polarons,^18^ i.e., quasiparticles comprised of a charge carrier and its self-induced lattice deformation. The latter process, enabled by strong electron–phonon coupling in the polar perovskite lattice, is thought to protect charge carriers from scattering with other charge carriers and phonons.^18^ In addition to increased carrier diffusion lengths,^19^ polaron formation may also dynamically compete with charge-carrier cooling on a (sub)picosecond time scales.^5,12,18,20^ Along these lines, dissimilar charge-carrier cooling rates in CsPbX_3_, MAPbX_3_ and FAPbX_3_ (MA = methylammonium, FA = formamidinium, X = Cl, Br, I) have been associated with differences in polaron stabilization energies.^5,12,18,21^ However, the notion that polaron formation slows down carrier cooling in perovskites^22^ seems at odds with the finding of fast (<50 fs) cooling in anatase TiO_2_ nanoparticles,^23^ possessing a similar electron–phonon coupling strength as LHPs, with Fröhlich coupling constants *α*_MAPbBr_3__ (=1.5 – 1.9) < *α*_TiO_2__ (≈2) < α_CsPbBr_3__ (=2.6 – 2.8).^21,23^ Moreover, reports on LHPs disagree on whether the organic cation accelerates^12,13^ or slows down cooling,^16^ and on the temperature dependence of cooling and polaron formation rates.^5,12,20,22^

The picture becomes even more complicated in perovskite NCs: next to further clarifying the role of phonons^24^ and composition,^25^ the possibility of size-dependent cooling rates arising from quantum confinement (leading to a sparser electronic DOS) and dielectric confinement (in the solvent/ligand environment) as well as ligand chemistry need to be addressed. While the description of confinement effects may borrow insights from the extensively studied II–VI, IV–VI, and III–V semiconductor NCs,^26−33^ e.g., the discussion of “phonon bottlenecks” (postulated slow cooling via LO phonon emission due to a mismatch of energy-level spacing and phonon energy), it remains unclear to which extent such models can be applied to polar and ionic semiconductors with a possibly reduced probability of scattering between charges and phonons via polaron “protection”.^5,21,22^

Several important recent findings have both fueled the charge-carrier cooling debate and complicated the assessment whether cooling can be slowed down in perovskite NCs: (i) cooling rates strongly depend on excitation density: cooling in perovskites may be significantly slowed down at high excitation density,^13,16,25^ due to overheated phonon modes (of similar importance as in many other polar semiconductors, e.g., GaAs), upconversion of acoustic to optical phonons, and a low thermal conductivity in perovskites; this requires “intrinsic” cooling rates of perovskites to be measured at low excitation densities; (ii) cooling rates are inconsistently defined: several ultrafast studies, including optical-pump THz-probe spectroscopy^12,34−36^ and the transient-absorption (TA) spectroscopy reported here and elsewhere^25,37^ clock the arrival time of cold carriers at the band edges; pump-push-probe spectroscopy is a variation on the theme, with altered IR oscillator strength of cold charges as the descriptor;^13,38^ in contrast, other studies employing time-resolved photoluminescence (PL) or TA spectroscopy monitor the decay of the hot population and the carrier “temperature” via fitting the high-energy tail using a Fermi–Dirac or Boltzmann distribution, an approximation which becomes invalid in strongly confined systems;^37^ finally, in femtosecond time-resolved two-photon photoemission (TR-2PPE) measurements^20^ as well as computational studies,^39−41^ the cooling rate was defined via the time evolution of the mean excess energy; in conclusion, comparisons of reported cooling rates are only meaningful if the same definition of cooling has been applied; (iii) charge-carrier- and state-resolved cooling has been elusive, as optical excitation and detection cannot easily discriminate electrons and holes, and the high and almost featureless DOS in perovskites essentially precludes a direct monitoring of the population of higher-energy states; (iv) while in CdSe NCs, control over the interaction of charges and phonons has been achieved via size confinement,^42,43^ ligand chemistry,^41^ or via tuning of the polar coupling through orbital symmetry and electron occupation,^42^ similarly detailed studies in perovskite NCs are still lacking.

Finally, despite new insights from recent discussions of the role of polaron formation rates^20^ and stabilization energies^21^ in LHPs, their respective relevance to charge-carrier cooling remains poorly understood even in bulk: for example, if polaron formation would compete with cooling, then overall cooling rate constants should increase with decreasing initial excitation energy since charge carriers have less time to get dressed as a polaron and thus prolong the cooling process. However, currently, such a correlation is not unambiguously supported by experiments.^5,12,20^ In this respect, it is important to note that previous models essentially rely on the harmonic and “time-averaged” polaron picture formulated by Fröhlich,^44^ Feynman^45,46^ and Osaka.^47^ The importance of a dynamic picture and anharmonicity has been identified by Bonn et al.^48^ who suggested that Fröhlich polaron transport additionally experiences “dielectric drag” induced by A-site cation rotations and rattling.

Here we take the first steps toward a truly time-dependent description via ab initio molecular dynamics (AIMD) supported by state-of-the-art ultrafast PL and TA spectroscopy. Time-domain simulations and experiments reveal that excited-state dynamics are governed by lattice vibrations, i.e., phonons, mediating strong time-dependent fluctuations of the electronic band structure. This dynamic picture provides an alternative explanation for the slow electron cooling close to the conduction band: without the need to invoke polaron formation, a slow final stage of the electron cooling process arises from suppression of intraband transition rates close to the conduction band edge. Our time-domain description allows us to address open questions regarding charge-carrier cooling in perovskite NCs, particularly the role of phonons and the dependence on the NC size. To this end, we extend the typically studied size regime, from the weak-to-intermediate confinement regime (4.1–12.3 nm, via ultrafast PL and TA spectroscopy), down to the intermediate-to-strong confinement regime (0.8–4.0 nm, via atomistic density-functional theory (DFT) calculations). Phonon-mediated cooling successfully explains three key experimental and computational findings: (i) a weak size dependence of cooling rates, due to a similar phonon DOS for NCs larger than 1.7 nm; (ii) comparable cooling rates of electrons and holes, in line with both conduction and valence band states predominantly formed by orbitals derived from the lead-halide sublattice; (iii) intraband gaps near the band edges modulated at frequencies of ∼15, ∼80, and ∼140 cm^–1^, corresponding predominantly to bending and stretching modes of the PbBr_6_^4–^ cage.^21^

Figure 1a depicts our structural model of a 2.9 nm CsPbBr_3_ NC, with Cs and Br atoms terminating the surface to emulate a surface capping of cations (e.g., oleylammonium, didodecyldimethylammonium) and anions (e.g., bromide, oleate, phosphonates). As shown recently,^49,50^ such a model (Cs_200_Pb_125_Br_450_ in case of a 2.9 nm NC) successfully describes the most common experimental observations of luminescent CsPbBr_3_ NCs, at a minimum computational cost. From the projected electronic density of states (PDOS) displayed in Figure 1b, we infer that the band-edge orbitals are mainly formed via Pb and Br atomic contributions, with Pb dominating the conduction band and Br the valence band, respectively. Cs contributes significant orbital character only to higher energy states, i.e., at about >0.5 eV excess energy. We furthermore verified that the electronic structure is devoid of localized midgap (trap) states in the displayed energy range.

**Figure 1:**
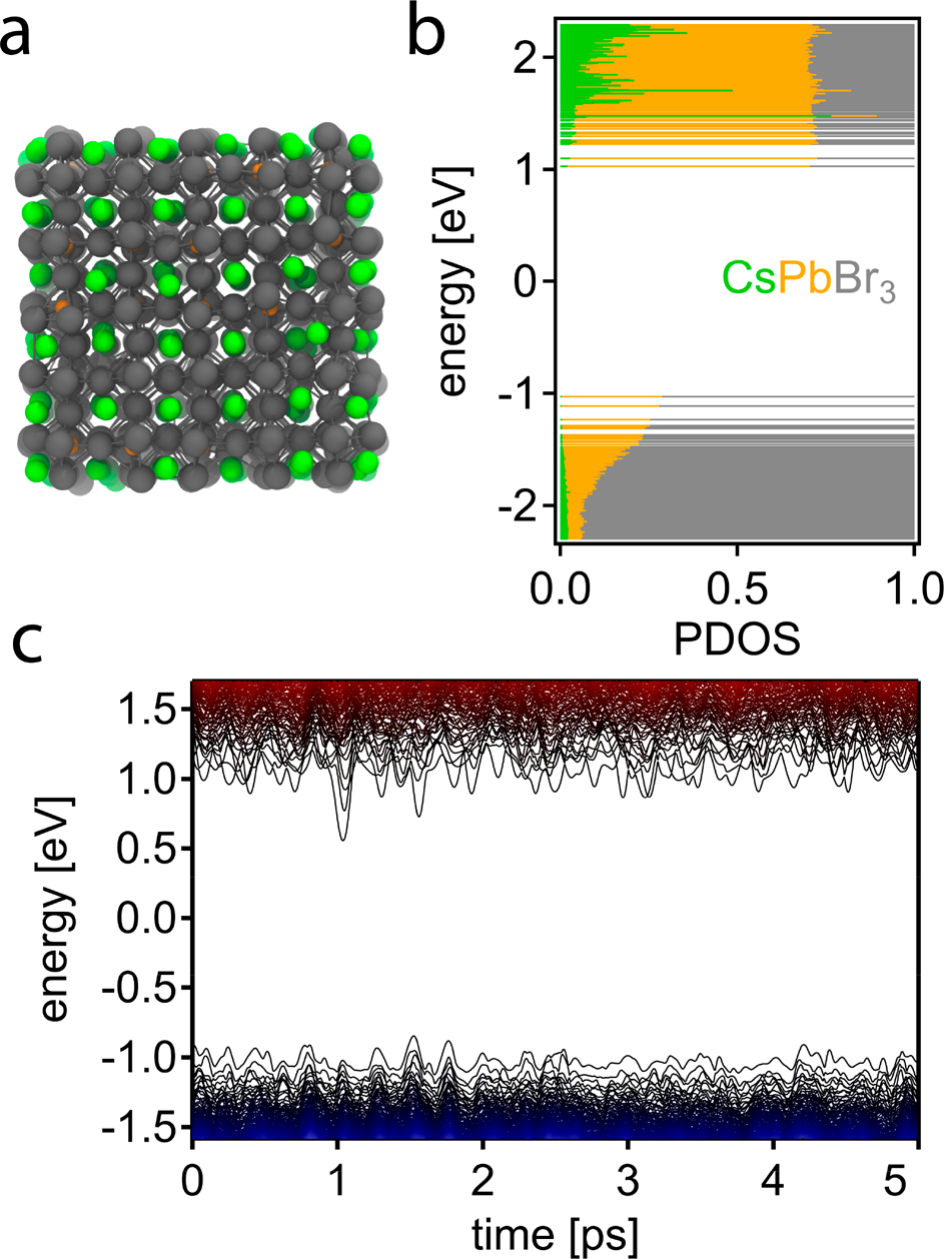
CsPbBr_3_ NC of 2.9 nm diameter. (a) Structural model, with Cs, Pb, and Br atoms depicted in green, orange, and gray, respectively. (b) PDOS per atom type. (c) Time-dependent Kohn–Sham orbital energies during an MD simulation of 5 ps.

After having determined the ground-state electronic structure, see Figure 1b, we follow its adiabatic time-dependent evolution in Figure 1c via AIMD simulations performed within a canonical ensemble at 300 K, employing the computational framework previously developed for CdSe,^51^ PbSe,^51^ and PbS NCs^41^ (see ref (41) and Supporting Information for details). Briefly, for each time step of the nuclear trajectory of the AIMD, we compute the adiabatic electronic structure at the DFT level of theory. In other words, we assume that the ground-state trajectory at room temperature also samples the excited-state potential energy surface. Owing to the soft nature of the perovskite lattice, the constituent atoms (especially the lighter Br and Cs) undergo large displacements from their equilibrium positions, translating into significant fluctuations of the electronic structure. Note that the energies of molecular orbitals fluctuate by several times *k*_B_*T*, i.e., ≫0.026 eV, and energy levels frequently cross in time (see also Figure S3 for NCs of different sizes). Such drastic dynamics of the electronic structure, a clear manifestation of strong electron–phonon coupling, raises important questions regarding charge-carrier cooling, such as what is the time scale and mechanism of charge-carrier cooling? Is the cooling rate different for electrons and holes? Also, related to discussions of phonon bottlenecks in confined systems,^13,18,52,53^ is there a size dependence for perovskite NCs?

To address these questions, we will now study charge-carrier cooling in colloidally dispersed CsPbBr_3_ NCs experimentally, via femtosecond PL and TA spectroscopy. Monodisperse CsPbBr_3_ NCs were synthesized according to a procedure laid out by Maes et al.,^54^ see Supporting Information for details. Figure 2a shows the linear absorption spectrum of the different sizes used. Given an estimated Bohr radius of 7 nm,^1^ they range from strongly confined 4.1 nm NCs to weakly confined 12.3 nm NCs, the former matching with the largest size used in the theoretical calculations. After photoexcitation, we study charge cooling in these colloidally dispersed NCs via ultrafast PL^55,56^ (see Figure 2b) and TA spectroscopy (see Figure 2c), respectively. While the former monitors the photoinduced rise of the PL intensity and the latter the change in absorbance Δ*A*, respectively, both techniques feature a time resolution of ∼150 fs, broadband detection, and tunable excitation with well-defined excess energy to the band gap. The measured Δ*A* signal originates from Pauli blocking (bleach) and spectral shifts, sometimes denoted as band gap renormalization in bulk materials. Importantly, the signal is composed of contributions from either electrons or holes. In contrast, PL measures the radiative recombination of electron–hole pairs implying that a signal can only be observed if both electron and hole occupy the relevant energy levels involved in the transition. As such, combining both PL and TA might elucidate differences in the cooling of electrons and holes.

**Figure 2:**
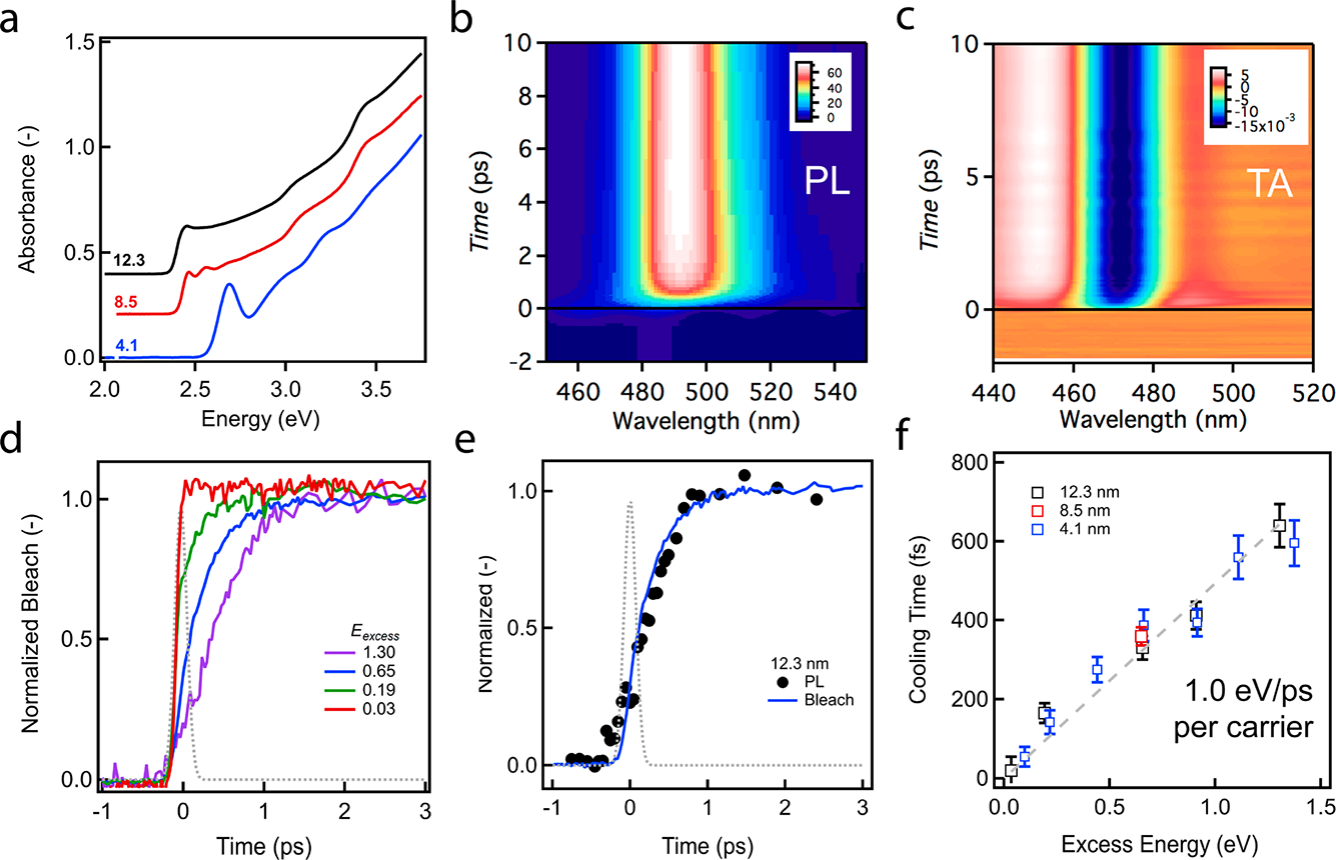
Overview of cooling experiments on CsPbBr_3_ NCs in the single-exciton regime, i.e., ⟨*N*⟩ ≪ 1. (a) Absorption spectra of 12.3 (black), 8.5 (red), and 4.1 (blue) nm NCs dispersed in *n*-hexane. Photoexcitation of 4.1 nm NCs with 0.65 eV excess energy, probed via ultrafast broadband PL (b) and TA spectroscopy (c), respectively, as a function of pump–probe time delay and probe energy. (d) Energy-integrated band-edge bleach, normalized at 3 ps, for the 12.3 nm NCs after photoexcitation with different excess energies *E*_excess_ (in eV). (e) Ingrowth of the energy-integrated TA band-edge bleach (solid blue) and PL intensity (black dots), normalized at 3 ps after photoexcitation with 0.65 eV excess energy. The dashed gray profiles in panels d and e indicate the pump–probe convolution giving a time resolution of 126 fs. (f) Extracted exciton cooling time from the deconvolution of traces shown in panel e for varying excess energy and particle sizes. A size-independent exciton cooling rate (i.e., electron and hole combined) of 2.02 eV ps^–1^, i.e., 1.0 eV ps^–1^ carrier^–1^, is obtained from the linear fit with zero offset (dashed gray line).

We note that photoexcitation in NCs always creates an electron–hole pair, a combination that can fuse to form a Coulombically bound exciton, which might have different properties than isolated charges. It was shown, however, that excitons dissociate rapidly in LHPs due to the limited binding energy compared to room temperature.^57^ The average number of absorbed photons per NC, often denoted as ⟨*N*⟩, is kept sufficiently low (⟨*N*⟩ ≪ 1) in both TA and PL experiments to avoid nonlinear effects such as optical gain, Auger recombination, and high-density phonon bottlenecks, all of which were observed earlier on similar NCs and in bulk perovskite materials.^58−60^ This regime also corresponds to the calculations presented further, where only single charges are present in the NCs.

Since spectral shifts around the band gap cloud the interpretation of the Δ*A* and PL signal as a direct metric for the population at that edge, we spectrally integrated the Δ*A* and PL signals, see Figure S2. For 12.3 nm NCs, Figure 2d shows the resulting kinetic traces of the ingrowing bleach, i.e., the band-edge population normalized at 3 ps, for different excess energies (*E*_excess_) relative to the band gap. Note that the *E*_excess_ defined here contains both electron and hole excess energy. Upon increasing *E*_excess_, we observe a slower ingrowth corresponding to the increased time carriers need to dissipate the larger excess energy. For *E*_excess_ = 0.65 eV, Figure 2e compares the ingrowth of the absorption bleach to the ingrowth of the integrated PL signal under similar excitation conditions. The almost perfect match indicates that electrons and holes cool at equal rates in these CsPbBr_3_ NCs. Figure 2f shows the resulting cooling times for all sizes, allowing three conclusions: (i) cooling times appear size-independent in the range of ca. 4–12 nm; (ii) the cooling time depends about linearly on the excess energy, indicating a fixed electron–hole pair energy dissipation rate of 2.02 eV/ps; (iii) the similarity of TA and time-resolved PL kinetics indicate that both electrons and holes cool on equivalent time scales, and we derive a dissipation rate of about 1.0 eV/ps per carrier.

We note that the observed excess-energy-independent dissipation rate is incompatible with a picture in which polaron formation efficiently competes with cooling.^20^ In the latter case, the dissipation rate should decrease for high initial excess energy, since cooling would be slowed down once the polaron has formed.^5^ The absence of such a trend in our data suggests that polaron formation is not (the only mechanism) responsible for slow cooling in CsPbBr_3_ NC. To understand this potentially surprising result, we may recall that polarons in CsPbBr_3_ are large, i.e., energetically weak, with reported formation energies on the order of 0.01–0.15 eV, changes to the Pb–Br bond length by ∼1%, bond angles altered by ∼10°, and polaron sizes of ∼3 nm.^21^ In light of these moderate (time-averaged) quantities, the phonon-mediated time-dependent fluctuations of the electronic structure (see Figure 1c and the discussion below) by up to 0.5 eV are large and likely influencing the cooling rates as well. Consequently, cooling may not only be determined by the relative rates of charge-carrier cooling and polaron formation but instead/additionally by the strength of electron–phonon coupling.

To describe cooling within a dynamic (i.e., time-domain) picture, and to obtain atomistic insights into the apparent indifference of electron and hole cooling rates as well as the observed size independence, we performed nonadiabatic molecular dynamics (NAMD) simulations on CsPbBr_3_ NCs from 0.8 to 4.0 nm (see Supporting Information for details). As in several other works on condensed-phase systems, we utilize the fewest-switches surface-hopping approximation with neglection of back reactions^61^ to simulate carrier cooling. Taking the dynamic electronic landscape in a 4.0 nm large Cs_490_Pb_343_Br_1176_ NC as a starting point (see Figure 3a), we initially excite either an electron or a hole to orbitals 0.5 eV above the respective band edges and subsequently follow the relaxation toward the band edges, indicated schematically by the white arrows in Figure 3a. Driven by large nuclear displacements at 300 K, the heavily time-dependent conduction band electronic structure leads to complicated relaxation patterns of the absolute energy of the electron, see Figure S4 in the Supporting Information. However, referencing the electron’s energy or hole’s energy to the conduction band minimum (CBM) or valence band maximum (VBM), respectively, at each point in time, allows us to follow the more insightful relaxation of the “excess energy” per charge carrier, see Figure 3b and Figures S4 and S6.

After an initially rapid cooling stage both for electrons and holes, in line with the similarity of PL and TA data, the subsequent cooling stage from about 1 ps proceeds more slowly: while the majority of the holes has quickly reached the band edge, this does not hold for the electrons, see Figure 3c. By fitting Maxwell–Boltzmann distributions to each of the charge populations, we find that holes have cooled within 2 ps to within 50 meV of the VBM, while even at the end of our time window at 4 ps, electrons remain excited in orbitals about 150 meV above the CBM, i.e., about 6 times *k*_B_*T*, see Figure S5 and Table S1. Such “persistent energetic electrons” have been reported experimentally before in MAPbI_3_, where electrons with 250 meV excess energy decayed with a time constant as large as 100 ps.^5^ Our results for the CsPbBr_3_ NC suggest that slow electron cooling in perovskites close to the CBM may be more general and at play even in the low carrier-density regime (one charge per NC) studied here.^58^ However, the amount of retained excess energy may depend on the specific halide,^13^ A-site cation,^13,25,37^ or (nano)size of the crystal.

**Figure 3:**
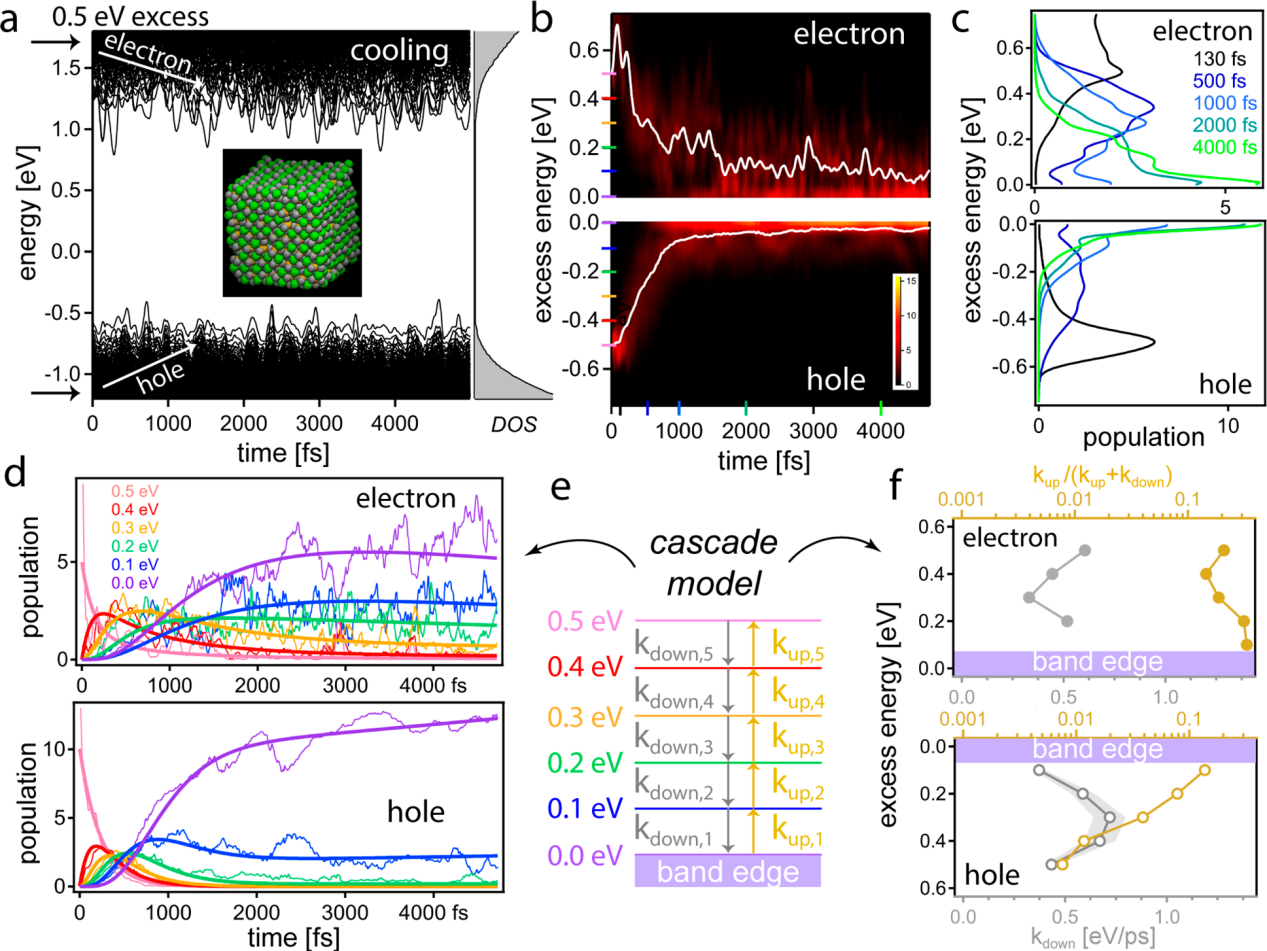
Charge-carrier cooling in a CsPbBr_3_ NC of 4.0 nm diameter. (a) At time zero, either one electron (top) or one hole (bottom) is placed at 0.5 eV above the respective band edge to trigger charge-carrier cooling along the time-dependent electronic structure at 300 K. The time-averaged DOS is depicted in the right panel. (b) Electron (upper panel) and hole cooling (lower panel), visualized via the statistically averaged populations (false-color images, see also Supporting Information) and average excess energy (white lines) as a function of time and excess energy above the band edge. (c) Selected “vertical” snapshots of the respective populations at various time delays, color-coded as the time markers in panel b. (d) Selected “horizontal” snapshots of the respective kinetics at various excess energies, color-coded as the energy markers in panel b. Thin lines represent data and thick lines the fits obtained in the cascade model in panel e. (e) Cascade model with both downward (relaxation) and upward (re-excitation) transitions between energy levels arbitrarily spaced by 0.1 eV. (f) Fit results of the cascade model, displaying the downward rates (gray markers and vs linear bottom axes) and the contribution of the upward rates to the total rates (ochre markers and vs logarithmic top axes).

To aid the comparison to the variety of reported ultrafast studies, we plot our data in two different representations: (i) energy-resolved populations at selected snapshots in time (see Figure 3c), which may be compared to, e.g., femtosecond time-resolved two-photon photoemission (TR-2PPE) measurements,^20^ and (ii) time-resolved kinetics at selected excess energies (see Figure 3d), which may be compared to, e.g., TA and time-resolved PL, as presented above, or PPP studies. The latter techniques monitor the arrival time of cold charges at the band edges, corresponding to the traces at 0–0.1 eV excess energy in Figure 3d. Band edge arrival times on the order of 1 ps for both the electron and hole are in qualitative agreement with our own TA results (∼0.5 ps for 0.5 eV excess energy per carrier, see Figure 2d) and those of other groups.^14,25^ An even more quantitative agreement is found between the higher excess-energy transients in Figure 3d and those deduced from a TR-2PPE study by Evans et al.^20^ Alternatively, we may monitor the average excess energy (white lines in Figure 3b), observable, e.g., in TR-2PPE measurements,^20^ which can be fitted with the decay model proposed by Prezhdo et al.,^62^ extended by a biexponential component (see Supporting Information). A similar biexponential fit has been employed before^16^ to describe the conceptually related cooling model accounting for scattering with LO and acoustic phonons.^24,59^ For the initial cooling stage at (within the first ∼1 ps and at >∼0.1 eV excess energy), we find cooling time constants *t*_cool,e_ = 0.69 ps for the electron and *t*_cool,h_ = 0.75 ps for the hole, corresponding to rate constants of 0.72 eV/ps and 0.67 eV/ps, respectively. This is in excellent agreement with the 0.64 eV/ps electron cooling rate found in a TR-2PPE study^20^ on CsPbBr_3_ single crystals and the 0.8 eV/ps charge-carrier cooling rate found in THz experiments on CsPbBr_3_ NCs,^34^ but a factor 1.5 and 2 lower than the cooling rates in CsPbBr_3_ NCs determined via TA in this and a previous^25^ study, respectively. We suggest that the small discrepancy with the latter studies may be accounted for when also including spin–orbit coupling in the calculations^63^ and the fact that cooling rates in the TA studies were defined via band-edge arrival times and fitted with single-exponential kinetic models. Overall, we observe rapid initial electron and hole cooling, slow electron cooling within 0.1 eV of the conduction band edge, confirm the ballpark of previously obtained cooling time constants, but reiterate (see above) the importance of referring to the same metrics when comparing the various cooling time regimes reported in literature.

To start rationalizing the data, we now drastically simplify the complex time-dependent electronic structure in Figure 3a and fit the kinetic traces in Figure 3d with a cascade model comprised of six static levels between 0 and 0.5 eV excess energy, arbitrarily spaced by 0.1 eV, see Figure 3e. As we will show now, this simplification (see Supporting Information for a discussion of applicability) captures the observed fast initial and slow subsequent relaxation. Specifically, we can now fit the electron and hole cooling kinetics in Figure 3d when allowing both relaxation (*k*_down,i_) and re-excitation (*k*_up,i_) transitions between adjacent levels, representing “cooling” and “heating”, respectively. Figure 3f summarizes the fit result: at all excess energies, the downward rates are similar for the electron (on average 0.47 eV/ps) and hole (on average 0.60 eV/ps). In contrast, the contribution of the re-excitation rate to the total rate (*k*_up,i_/(**k**_u__p__,_*_i_* + *k*_down,i_)) significantly increases toward the band edges: for the hole from less than 1% at 0.5 eV up to 14% at 0.1 eV excess energy, and for the electron from 21% at 0.5 eV to 33% at 0.1 eV excess energy.

Re-excitation rates approaching relaxation rates straightforwardly explain the large population of higher-energy states observed after ≥1 ps, i.e., after completion of the initial cooling stage, especially in the case of electrons. We note here that we can exclude Auger heating as a possible origin,^3,15^ as we study the single charge-carrier picture, differing from the exciton and other many-particle excitations studied upon optical excitation. Instead, persistent energetic electrons from re-excitations appear due to the phonon-mediated fluctuations of the band structure, culminating in frequent state crossings close to the CBM (see Figure 3a). Upon a state crossing, the carrier acquires excess energy if it does not immediately relax to the new lower-energy state. This thus corresponds to a “heating” event in the static cascade model. Its likelihood increases with the occurrence of state crossings. Phonon-mediated energy fluctuations naturally also explain the observed variations in overall cooling rates: whereas higher-energy states (∼0.5 eV above the band edges) are more coherent and facilitate the initial rapid cooling stage, the final stage (within ∼0.1 eV of the band edges) proceeds slower due to incoherently modulated state energies, enabling re-excitation to higher-energy states via state crossings; the latter is more pronounced for the conduction band-edge states than for the valence band-edge states, yielding slower electron cooling close to the CBM.

Accepting the qualitative suggestions by the cascade model, we now present a comparison of experiment and theory, attempt to further understand possible size effects (affecting both nuclear and electronic degrees of freedom), and resolve the spectrum of involved phonon modes. Remarkably, contrasting a large number of reports on size-dependent excited-state dynamics in II–VI and IV–VI semiconductor NCs,^28^ only a few pioneering studies reported on size-dependent charge-phonon couplings^64−66^ and their involvement in charge-carrier cooling.^67^ Elucidation of phonon-driven excited-state dynamics is even more needed in the soft perovskite NCs. Computationally, we compare seven NCs from 0.8 to 4.0 nm edge length (see structural models in Figure 5a), and experimentally from 4.1 to 12.3 nm (see Figure S1). To the best of our knowledge, this constitutes the first report with matching computational and experimental sizes, and the most thorough (combined) size dependence so far.

Figures 4a–c demonstrate the close match of experiment and theory: first, and following previous reports,^16,58,59^ we identify the TA bleach to the blue of the band gap as a proxy for warm charge carriers (see Figure 4a). In Figure 4b, we then monitor the cooling kinetics of warm carriers in 4.1 nm NCs by plotting the decay of the bleach fwhm. While such analysis has been applied previously, we acknowledge its limitations (see Supporting Information for a detailed discussion) and regard the transient “excess” fwhm (with respect to the long-term limit, e.g., at 1 ns) as a lower bound for the real electron and hole excess energy. As the rise of the band gap bleach above, the transient fwhm can be fitted with a biexponential decay, representative of a two-stage cooling process. The slow stage involves 10 meV excess fwhm decaying over 10 ps back to thermal equilibrium (∼60 meV fwhm at 1 ns). Figure 4c shows that the computational cooling data for electrons in essentially equally sized 4.0 nm NCs (see also Figure 2) can likewise be fitted with biexponential decay (see above and Supporting Information for details), yielding an amplitude of ∼100 meV with the experimentally found long time constant of 10 ps. In other words, warm electrons within 100 meV of the CBM are persistent in time, decaying only at a rate of 0.01 eV/ps back to thermal equilibrium. With the TA and computational results as lower and upper bounds, respectively (see Supporting Information for details on the CBM energy referencing), we conclude that the slow cooling stage involves electrons that are between 10 and 100 meV warmer than thermal equilibrium, i.e., described by a carrier temperature between ∼400 and ∼1500 K, and persisting for ∼10 ps.

**Figure 4:**
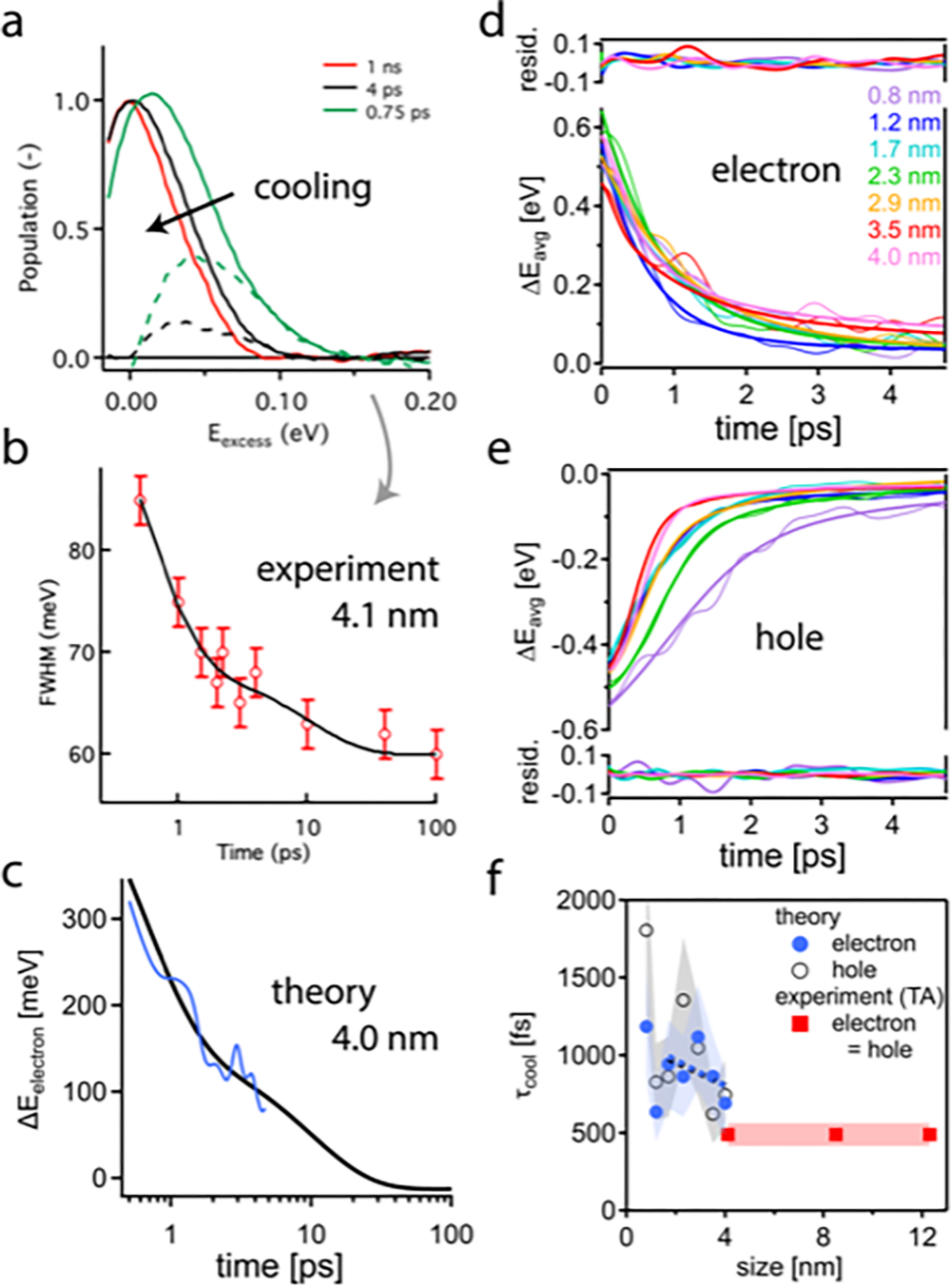
Size-dependent electron and hole cooling in CsPbBr_3_ NCs from 0.8 to 12.3 nm, with an initial excess energy per charge carrier of 0.65 and 0.5 eV in the TA experiment and NAMD calculations, respectively. (a) Normalized TA bleach spectra in 12.3 nm NCs 0.75 ps, 4 ps, and 1 ns after photoexcitation, depicted by green, red, and black solid lines, respectively. The transient excess energy is monitored as a bleach at the blue side of the peak and visualized as an excess bleach at 0.75 and 4 ps (with respect to cooled charges at 1 ns) by green and black dashed lines, respectively. (b) Decay of the fwhm of the TA bleach in panel a, as a proxy for hot charge carriers, in 4.1 nm NCs (red open circles), and biexponential fit (black solid line). (c) Electron excess energy for a similarly sized 4.0 nm NC (red solid line) obtained via NAMD simulations and biexponential fit (black solid line). (d) Decay of the average excess energy (Δ*E*_avg_) of an electron; thin and thick lines represent computational data and corresponding fits, respectively, with the residuals depicted in the top panel. (e) Corresponding computational data, fits, and residuals for the hole. (f) Electron and hole cooling time constants per carrier extracted from the fits in panels d and e, respectively, along with the experimentally assessed time constants from the TA data in Figure 2f. Shaded regions depict the error margins, estimated to be ±15% and ±30% in experiment and theory, respectively. Black and gray dashed lines (depicting linear fits to electron and hole data between 1.7 and 4.0 nm) serve as a guide to the eye.

Figure 4d,e show the computed size-dependent cooling dynamics for the electron and hole, respectively, in CsPbBr_3_ NCs from 0.8 to 4.0 nm. Fits yield weakly size-dependent initial cooling time constants *t*_cool_ (see Figure 4f), consistent with a weak phonon bottleneck effect due to quantum confinement. Slightly slower initial cooling for smaller NCs confirms and extends previous results on strongly confined CsPbBr_3_^39^ and weakly confined MAPbBr_3_ NCs.^24^ Only the smallest 0.8 nm NC departs from the weak size dependence, as here both electron and hole cooling proceed significantly slower, in 1.4 and 1.8 ps, respectively, yielding an electron–hole pair cooling rate of 0.63 eV/ps. However, we note that this particular NC is barely larger than one CsPbBr_3_ unit cell; in fact, due to its ultrasmall size, the Cs:Pb:Br stoichiometry strongly deviates from 1:1:3 and essentially resembles the Cs_4_PbBr_6_ motif. When excluding the strongly off-stoichiometric 0.8 and 1.2 nm NCs, the average initial cooling time constants *t*_cool_ for CsPbBr_3_ NCs between 1.7 and 4.0 nm are 0.90 and 0.93 ps for the electron and hole, respectively, corresponding to a size-averaged initial electron–hole pair cooling rate of 1.10 eV/ps.

In the second and slower cooling stage, electrons reach thermal (quasi-) equilibrium at the CBM via dissipating the last 20–100 meV excess energy over a time scale of about 10 ps, for all sizes (see fits in Figure 4d). The size-averaged persistent electron excess energy amounts to 42 meV (or 800 K). The lack of a size dependence suggests that the slow cooling stage is not a result of quantum confinement and a phonon bottleneck due to a sparse DOS, as discussed for CsPbBr_3_ and MAPbBr_3_.^39^ Instead, as introduced above, we interpret the persistent warm electron population as a fingerprint of efficient phonon-mediated re-excitation in the vicinity of the CBM. Potentially, such an assignment may also be a candidate for (at least partially) explaining the persistent hot electrons (at up to ∼100 ps) found in bulk LHPs,^5,22^ i.e., materials lacking quantum confinement. In contrast to electrons, holes in our CsPbBr_3_ NCs do not feature such a clearly discernible slower cooling stage: the remaining excess energy after the initial cooling amounts to only 27 meV on average, i.e., about *k*_B_*T*, as expected for the “conventional” picture of a static, unperturbed band structure.

Singling out predominant phonon modes that mediate excited-state dynamics requires an inspection of the phonons DOS and the interaction of charges and phonons. For the perovskite NCs from 0.8 to 4.0 nm (see Figure 5a), the bottom panels in Figure 5b show that the phonon DOS is almost size-independent. Three LO phonon peaks at ∼80, ∼100, and ∼140 cm^–1^ can be discerned, related to Pb–Br–Pb bending and Pb–Br stretching,^21,68,69^ and a broad low-energy peak consistent with local polar fluctuations, e.g., Cs^+^-induced deformations of the PbBr_6_^4–^ cage, as previously observed via Raman spectroscopy.^68^ Only NCs equal or smaller than 1.2 nm exhibit significantly altered phonon spectra; this is similar to the case of CdSe, where a transition from bulk-like phonons to molecular-type vibrations has been observed at 2 nm, a size at which crystallites contain only a single complete zincblende unit cell.^70^ Altered phonon spectra for the smallest NCs are furthermore expected given their increasing off-stoichiometry which approaches the Cs_4_PbBr_6_ motif at 0.8 nm. Overall, the weakly size-dependent phonon DOS for NCs > 1.2 nm matches well the weakly size-dependent initial cooling rates of electrons and holes.

**Figure 5:**
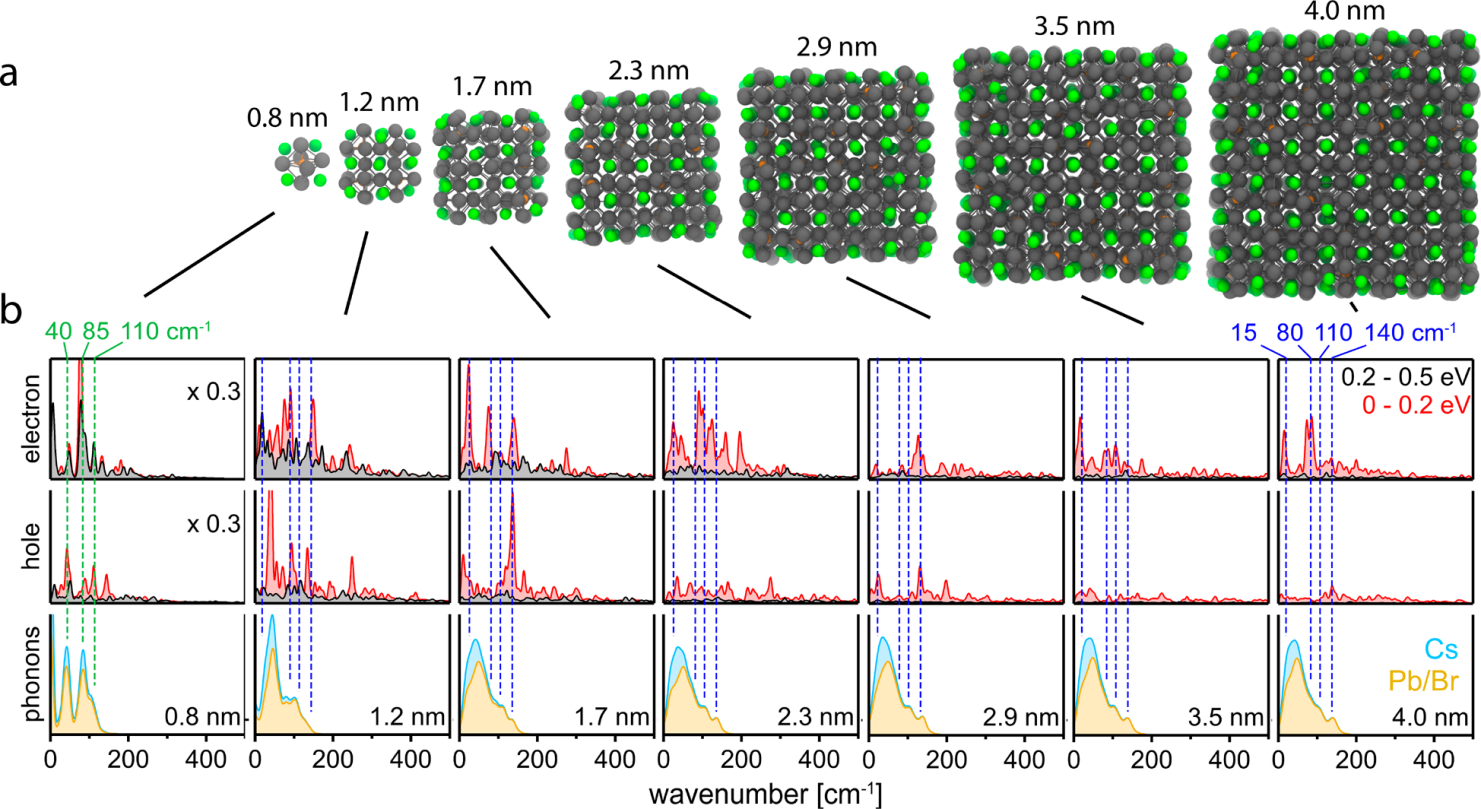
(a) Geometrical models of CsPbBr_3_ NCs from 0.8 to 4.0 nm, with Cs, Pb, and Br atoms depicted in green, orange, and gray, respectively. (b) Coupling of charge carriers and phonons: upper panel, phonon influence spectra for neighboring conduction band electronic states with excess energies averaged between 0 and 0.2 eV (red shaded area) and between 0.2 and 0.5 eV (black shaded area), respectively; middle panel, corresponding phonon influence for neighboring hole states in the valence band, with identical y-scaling as for the electron states; bottom panel, phonon DOS, normalized to the number of atoms per NC, with contributions from the lead bromide cage (ochre) and cesium (light blue). In NCs from 1.2 to 4.0 nm, the predominant phonon influence modes at about 15, 80, and 140 cm^–1^ (indicated by the blue dashed vertical lines) are attributed to Br–Pb–Br bending and Pb–Br stretching, respectively. In the 0.8 nm NC, the predominant modes at about 40, 85, and 110 cm^–1^ (indicated by green dashed vertical lines) match those of Cs_4_PbBr_6_.

The phonon-mediated coherence loss between neighboring intraband states, expressed here as a “phonon influence spectrum” for electrons and holes, is depicted in the upper and middle panels of Figure 5b, respectively (see Supporting Information for calculation details). Briefly, the phonon influence spectrum is calculated as , where *C*(*t*) = ⟨Δ*E*(*t*)Δ*E*(*0*)*/*⟨Δ*E*^2^(0)) is the normalized autocorrelation function for an intraband transition of energy *E*_*ij*_ between neighboring states *j* = *i* + 1 and *i*, with Δ*E* = *E*_*ij*_ – ⟨*E*_*ij*_⟩ denoting the fluctuation with respect to the statistically averaged transition energy ⟨*E*_*ij*_⟩. At high excess energy (0.2–0.5 eV above the respective band edge), the phonon influence spectra are of low amplitude and almost featureless. This indicates that, for both charge carriers, the initial cooling proceeds via transitions between states whose energy difference fluctuates only a little, at least on the time scale of the cooling process. Such slight incoherence is consistent with thermally activated cooling rates^12^ due to momentarily lower energy separations (and thus increased adiabatic coupling) between intraband states. Along these lines, the significantly increased phonon influence for smaller NCs (see Figure 5b) should also yield enhanced nonadiabatic couplings and faster cooling. However, the opposite trend is observed, with a weak deceleration of cooling for very small NCs (see Figure 4c). We reconcile both results by acknowledging that phonon bottlenecks (due to a sparser DOS)^39^ likely annul the size dependence of the nonadiabatic couplings and even slow down cooling for very small NCs.

In the final cooling stage, i.e., within 0.2 eV of the band edge, the phonon influence increases markedly, and the decoherence between states occurs at frequencies covering and exceeding the entire spectral range of the phonon DOS. Predominantly involved are the Pb–Br–Pb bending and Pb–Br stretching vibrations, at around 80, 110, and 140 cm^–1^, next to low- and high-frequency contributions. Whereas the involvement of LO phonons is intuitive, higher frequencies may originate from combination bands and overtones, and lower frequencies may be a signature of difference frequencies, surface modes, or Cs^+^-induced local polar fluctuations deforming the PbBr_6_^4–^ cage.^68^ Smaller NCs exhibit sharper features in the phonon influence spectrum, in line with the sharper “molecular-type” vibrations observed in the phonon DOS (see bottom row in Figure 5b). Interestingly, the relative increase of the phonon influence at low energy (within 0.2 eV of the band edge) as compared to high energy (0.2–0.5 eV above the edge) is more pronounced for larger NCs. Together with a larger number of state crossings stemming from a tighter energy level spacing, it is conceivable that larger NCs can sustain persistent energetic carriers at rather high excess energies. While further work will be required to investigate the matter, our phonon influence spectra suggest that not only thermal broadening^41^ (involving the CBM-VBM interband transition) but also charge-carrier cooling (involving intraband transitions) is governed by strong charge-phonon interactions in CsPbBr_3_ NCs, consistent with the thermal activation of both processes.^12,71−73^ A tempting future extension of our work would be to make use of the obtained atomistic insights in CsPbBr_3_ NCs and study charge-phonon coupling in related material systems. For example, we anticipate that it could be a worthwhile endeavor to attempt controlling the observed phonon influence in LHP NCs via appropriate tuning of the composition, stoichiometry, or surface termination.

In summary, we address open questions regarding the rate and origin of slow charge-carrier cooling in LHPs by studying CsPbBr_3_ NCs via both experimental (ultrafast laser spectroscopy) and computational chemistry methods (NAMD). Studying a very broad combined size regime, from 0.8 to 12 nm, i.e., covering the strong to weak confinement regime, we establish that cooling proceeds initially at about 1 eV ps^–1^ carrier^–1^, decreasing only weakly with decreasing size, and roughly independent of excess energy and carrier type (i.e., electron or hole). Within about 100 meV of the CBM, however, electron cooling is significantly slowed down, with rates decreasing by more than 1 order of magnitude. The agreement of our NAMD simulations with the recently reported persistent energetic electrons in LHP thin films is surprising: while the latter results have been suggested to arise from a competition between polaron formation and cooling, our NAMD simulations on perovskite NCs suggest that slow cooling close to the CBM may not require polarons and is, at least in part, a result of phonon-mediated fluctuations of the electronic structure. Spectral analysis shows that a wide spectrum of thermally populated phonons induces decoherence of the electronic structure, especially within about 0.2 eV of the CBM, and predominantly mediated by deformation, bending, and stretching modes of the PbBr_6_^4–^ cage at about 15, 80, 110, and 140 cm^–1^. Our study reiterates the importance of strong charge-phonon coupling in LHPs, yet emphasizes the dynamic nature of the interaction. Formulated in the time-domain, excited-state dynamics are intuitively tracked, and results can be directly compared to a variety of ultrafast spectroscopies. As our combined experimental–computational approach may be straightforwardly extended to other materials systems, we hope that it may aid the fundamental understanding of excited-state dynamics in polar semiconductors in general and accelerate the development of devices utilizing hot charge carriers.

## Supporting Information Available

Materials; nanocrystal synthesis; sizing analysis via TEM; time-resolved and spectrally resolved TA and PL data; TA bleach at high excess energy and a short time scale; computational chemistry methods; time-dependent electronic landscape; energy- and state-resolved electron cooling kinetics; Maxwell–Boltzmann fit to electron and hole populations from NAMD simulations; fit of the decay of the average excess energy in NAMD simulations (PDF)

## Abbreviations

AIMD: ab initio molecular dynamics
NAMD: nonadiabatic molecular dynamics
DFT: density functional theory
TA: transient absorption
PL: time-resolved PL
TR-2PPE: time-resolved two-photon photoemission
MEG: multiple exciton generation
LHPs: lead-halide perovskites
