# Supplementary material for: Phonon-Mediated and Weakly Size-Dependent Electron and Hole Cooling in CsPbBr3 Nanocrystals Revealed by Atomistic Simulations and Ultrafast Spectroscopy
Source: Nano Lett. 2020 Feb 12;20(3):1819–29. doi: 10.1021/acs.nanolett.9b05051 (PMC7997624; doi:10.1021/acs.nanolett.9b05051)
Supplement: Supplementary file 1 — nl9b05051_si_001.pdf [file nl9b05051_si_001.pdf]

Supporting Information belonging to:

Phonon-mediated and weakly size-dependent electron and hole cooling in $CsPbBr_3$ nanocrystals revealed by atomistic simulations and ultrafast spectroscopy

Simon C. Boehme,[† #*] *Stephanie ten Brinck,*[#] *Jorick Maes,* [□] *Nuri Yazdani,*[¶] *Felipe Zapata,*[&] *Kai Chen,*[‡] *Vanessa Wood,*[¶] *Justin Hodgkiss,*[‡] *Zeger Hens,* [□] *Pieter Geiregat,* [□] *Ivan Infante*[#, Δ,*]

[#] Department of Theoretical Chemistry, Faculty of Science, Vrije Universiteit Amsterdam, De Boelelaan 1083, 1081 HV Amsterdam, The Netherlands

[□] Department of Chemistry, Faculty of Sciences, Universiteit Gent, Krijgslaan 281, 9000 Gent, Belgium

[¶] Materials and Device Engineering Group, Department of Information Technology and Electrical Engineering, ETH Zurich, Zurich CH-8092 Switzerland

[&] Netherlands eScience Center, Science Park 140 (Matrix I), 1098 XG Amsterdam, The Netherlands

‡ The MacDiarmid Institute for Advanced Materials and Nanotechnology, Wellington, 6012, New Zealand; School of Chemical and Physical Sciences, Victoria University of Wellington, Wellington, 6012, New Zealand

Δ Department of Nanochemistry, Istituto Italiano di Tecnologia, Via Morego 30, 16163 Genova, Italy

* sboehme@ethz.ch ; ivan.infante@iit.it

† Present Address: Institute of Inorganic Chemistry, Department of Chemistry and Applied Biosciences, ETH Zurich, 8093 Zurich, Switzerland

Materials – Nanocrystal Synthesis

To synthesize the $CsPbBr_3$QDs, we adapted a synthesis by De Roo and Maes.[1,2] Cesium(Cs)-oleate was synthesized as a precursor for cesium (Cs). To this end, 0.407 g of Cs_2CO_3 (2.5 mmol, Sigma-Aldrich, 99%) was loaded into a 50 mL 3-neck flask along with 20 mL of 1-octadecene (ODE) (tech. 90%, Alfa Aesar) and 1.55 mL of oleic acid (OA) (5 mmol, tech. 90 %, Alfa Aesar) dried for 1 h at 120 °C under nitrogen, and then heated to 150 °C until all of the everything dissolved. As Cs-oleate is insoluble in ODE at room temperature, it has to be pre-heated before the injection. The above results in a final concentration of 0.116 M.

Next, 138 mg of $PbBr_2$ (0.376 mmol), Alfa Aesar, Puratronic 99.999% (trace metal basis) is loaded into a 25 mL three-necked flask and 7.5 mL of ODE is added. The cloudy suspension is heated under nitrogen at 120 °C for 30 min. Subsequently, 1 mL of both oleylamine (OLAm) (3 mmol), Acros Organics, approximate C_{18} content 80-90% and OA (3 mmol) is injected and the temperature is raised to 180 °C after the quick dissolution of $PbBr_2$. Now, 0.8 mL of Cs-oleate solution is injected and the resulting cloudy, yellow mixture is cooled with an ice bath, upon which the color changes to bright green. The crude synthesis mixture is centrifuged for 5 min at 3900 G and the colored supernatant is discarded. The precipitate is re-dispersed in 1.2 mL of n-hexane (VWR, technical) and again centrifuged. The precipitate is now discarded and the resulting supernatant is colloidally stable after addition of another 0.3 mL n-hexane. No special care was taken to dry solvents or surfactants. To the 1.5 mL of $CsPbBr_3$ dispersion, 40 μl of both OA and OLAm are added. Gradually making the solvent more polar with the aid of acetone (Fiers, acetone pure > $99.5%) makes it possible to split the polydisperse synthesis mixture into distinct fractions with a lower size dispersion. First, 1 ml of acetone is added and centrifuged at 1900 G for 2 min. The precipitate containing a broad range of large particles is discarded and now the size selective precipitation can be carried out on the supernatant. 125 μl of acetone is added and the resulting precipitate is dispersed in 0.5 mL n-hexane containing 1 vol% OLAm to ensure long-time colloidal stability. Repetitive addition of 125 μl of acetone, each time followed by centrifugation at 1900 G for 2 min, yields *ca.* 10 fractions of monodisperse $CsPbBr_3$ QD dispersions.

Sizing analysis via TEM

Sizes of select fractions were analyzed using transmission electron microscopy (TEM) which reveals excellent monodispersity for all sizes used, see Figure S1.

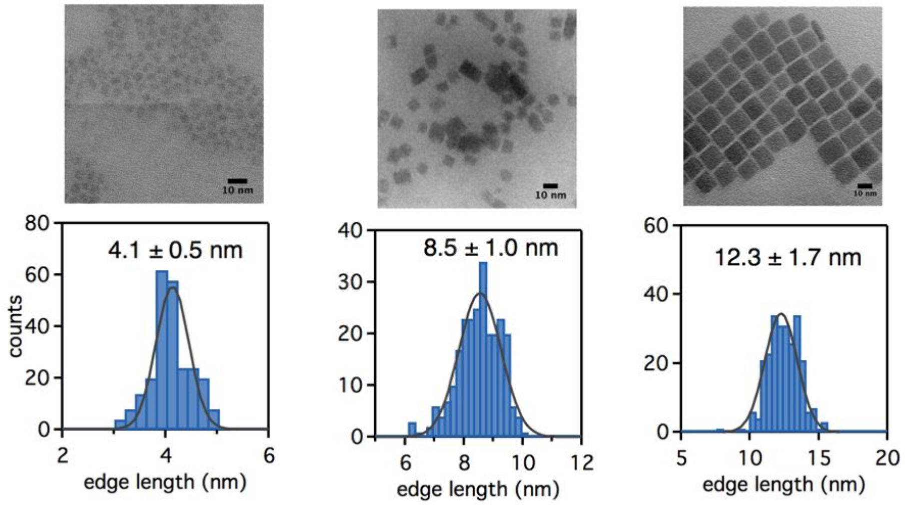

Figure S1: Sizing analysis of the $CsPbBr_3$ QDs using transmission electron microscopy (TEM).

Time- and spectrally resolved TA and PL data

Methodology - Transient absorption spectroscopy

Samples were excited using 110 femtosecond pump pulses with varying wavelength created from the 800 nm fundamental (Spitfire Ace, Spectra Physics) through non-linear conversion in an OPA (Light Conversion, TOPAS). Probe pulses were generated in a thin, translated, CaF_2 crystal using the 800 nm fundamental. The pulses were delayed relative to the pump using a delay stage with maximum delay of 6 nanoseconds, though we only focus on the cooling stage. The probe spectrum in our experiments covers the UV-VIS window from 350 nm up to 700 nm. $CsPbBr_3$ QDs were dispersed in an optically transparent solvent (n-hexane) and continuously

stirred to avoid charging or photo-degradation. The quantum yield before and after the measurements was comparable at a typical value of $\approx$ 65% measured using an integrating sphere.

The average number of photo-generated charge pairs at time zero, noted as $\langle N \rangle$, can be calculated from the photon flux J_{ph}, the cuvette length L and the nanocrystal absorption cross section at the pump wavelength $\sigma_a(\lambda)$:

$$\langle N \rangle = J_{ph}\sigma_a(\lambda)$$

The photon flux is calculated from the beam area, obtained through a Thorlabs CCD beam profiler, and defined as $A = 2\pi\sigma_x\sigma_y$ where σ_i is the standard deviation in the *i=x,y* direction. We can write the cross section at a given wavelength as the product of the intrinsic absorption coefficient at that wavelength and the volume of the NC:[3] $\sigma_a(\lambda) = \mu_{i,0}(\lambda)V_{QD}$. The volume is determined from the TEM analysis and the intrinsic absorption coefficient[3] $\mu_{i,0}$ of $CsPbBr_3$ QDs was determined in earlier work through a combination of elemental analysis and TEM, where Maes *et al.* showed that $\mu_{i,0}$ is a size-independent quantity with a value of: $\mu_{i,0}$ (335 nm) = 1.59 $\pm$ 0.05 10^5 cm^{-1}.[2] Since we are interested in the dynamics of single excitations, we work under conditions where $\langle N \rangle < 0.05$ for all samples and pump wavelengths reported.

<u>*Methodology - Ultrafast Luminescence Spectroscopy*</u>

For (ultrafast) photoluminescence (PL) spectroscopy, samples were measured using a cuvette with an optical path length of 1 mm and an optical density of ca. 0.1 at the exciton absorption peak to avoid strong re-absorption. The detection of the broadband PL on femtosecond

timescales was made possible by the transient grating technique by Chen *et al.*[4] A Ti:Sapphire amplifier system (Spectra-Physics Spitfire Ace) operating at 3 kHz generating 110 fs pulses was split into two parts. One part was frequency doubled to 400 nm using a BBO crystal, or converted to different wavelengths using on OPA (Topas, Light Conversion), and focused to a 70 μm^2 spot on the sample. The PL is collimated using a silver coated off-axis parabolic mirror and refocused on a polished slice of fused silica. The second part, *ca.* 40 μJ, of the 800 nm output was again split using a 50/50 beam splitter creating two *gate* beams that are focused on the fused silica plate with a crossing angle of approximately 8 degrees. The instantaneous grating generated by the interfering gate beams creates an instantaneous gate which is used to temporally resolve the decay over a broad wavelength range. The scatter of the pump beam was suppressed using a 430 nm long-pass filter and the pump polarization was set a magic angle relative to the PL collection. Spectra were averaged over 15000 shots at every time delay.

<u>*Data analysis*</u>

The integration boundaries for the TA and PL data analysis are determined based on the extension of the induced absorption at 490 nm. The latter originates from spectral shifting and dies out at 500 nm, right boundary, which is 30 nm red-shifted from the bleach maximum at E_g = 470 nm. We use this 30 nm also to define the short wavelength limit, *i.e.* at 440 nm (E_g-30 nm), resulting in a 60 nm (*ca.* 300 meV) integration window.

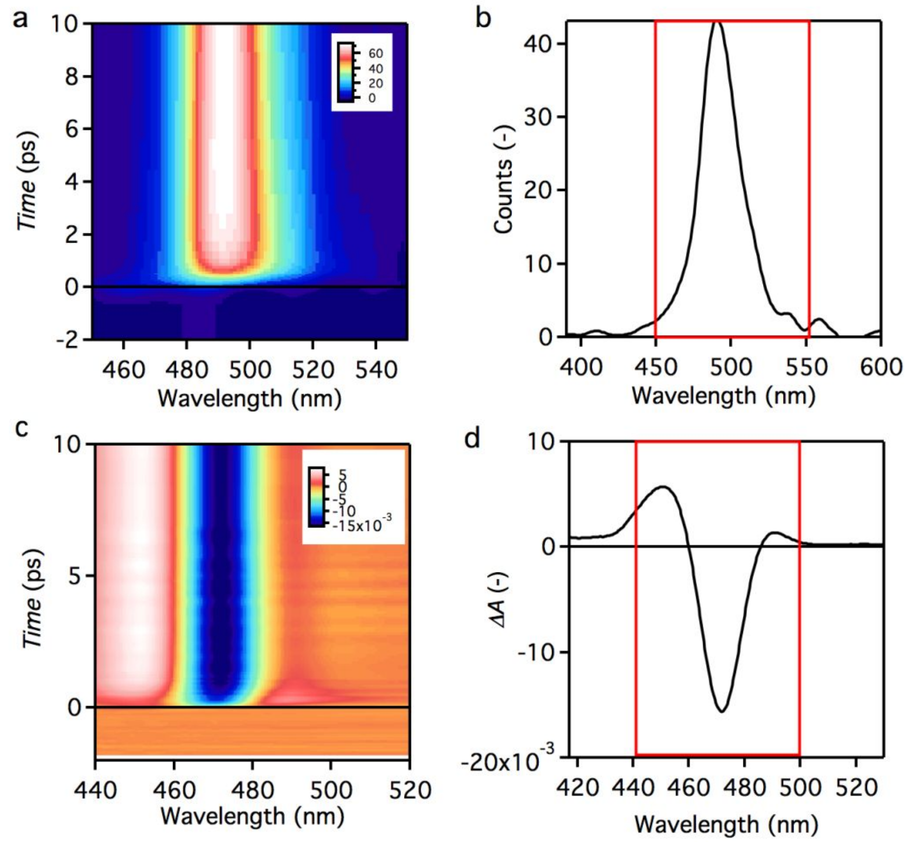

Figure S2: PL (top) and TA (bottom row) data analysis on 4.1 nm dots with 400 nm excitation. (a-b) Typical PL map and the defined integration boundaries in red. (c-d) Typical TA map and the integration boundaries used.

TA bleach at high excess energy and short time scale

A carrier population with excess energy should manifest itself in the TA spectra as a bleach component at high photon energy. From this excess bleach to the blue of the band gap, previous reports inferred the carrier temperature during cooling.[5,6] We should be wary however that the increased DOS at these high energies will dilute the effect of a sizable carrier population since TA probes only relative changes, *i.e.* population/DOS. Nonetheless, we do observe excess bleach at high energy when pumping with 330 nm, shown for the 12.3 nm dots in Figure 4a of the main text. We show the normalized bleach for 0.75 ps, 4 ps, and 1 ns. The dashed curves are the difference between the early-time spectra (0.75 and 4 ps) and the long-time spectra (1 ns) separately. For example, the dashed green curve is a measure for the excess population profile at 750 fs which seems to be centered around 50 meV with an asymmetric broadening up to 150 meV. With the limitations introduced above, we then take the (excess) FWHM (plotted in Figure 4b of the main text) as a lower bound of the true excess energy.

Computational chemistry methods

In our *ab initio* molecular dynamics (AIMD) simulations, both the nuclear trajectory and time-dependent electronic structure are obtained with the CP2K computational package. First, we generate the nuclear trajectory at 300 K (NVE ensemble, with a time step of 2.5 fs and a total length > 4 ps) after equilibration of the total energy (NVT ensemble). In the latter, we employ a CSVR thermostat, *i.e.* canonical sampling via velocity rescaling.[7] Subsequently, we obtain the time-dependent electronic structure for every point of the nuclear trajectory at the density functional level of theory.

To calculate the excited-state dynamics, we perform non-adiabatic molecular dynamics (NAMD) calculations using the QMFlows-NAMD package (recently introduced by F. Zapata et al.[8] and available via https://github.com/SCM-NV/qmflows-namd). For each step of the nuclear trajectory, the molecular orbital overlaps within about 0.7 eV of the band edges are utilized in a routine based on the PYXAID package.[9] The cooling of one electron (one hole) is initiated by placing the charge carrier into a molecular orbital 0.5 eV above (below) the conduction (valence) band edge. The subsequent relaxation towards the band edge is followed *via* the time-dependent state occupations $n_i(t)$ by evaluating the non-adiabatic couplings $d_{i,j} = -\hbar \left\langle \varphi_i(t) \middle| \frac{\partial}{\partial t} \middle| \varphi_j(t) \right\rangle$ between to neighboring states i and j at each time step of the nuclear trajectory. Stochastic sampling is obtained under the fewest switches surface hopping approximation with neglection of back reactions (FSSH-NBRA) previously employed[10] and described in detail by the Prezhdo group.[11] Detailed balance is ensured by weighting transitions upward in energy with a Boltzmann factor. After averaging decay traces across 100 initial conditions (with 250 stochastic realizations

each) differing a few hundreds of fs in time and about hundred meV in excess energy, the occupation weighted density of states $p(E,t) = \sum_i n_i(t)\,\delta\left(E_i(t) - E_{ref}(t)\right)$ is obtained with respect to a reference energy $E_{ref}(t)$, chosen to be the conduction and valence band edge, respectively, in the false-color plots in Figure 3b of the main text.

The phonon density of states has been obtained via the TRAVIS analyzer.[12,13]

Time-dependent electronic landscape

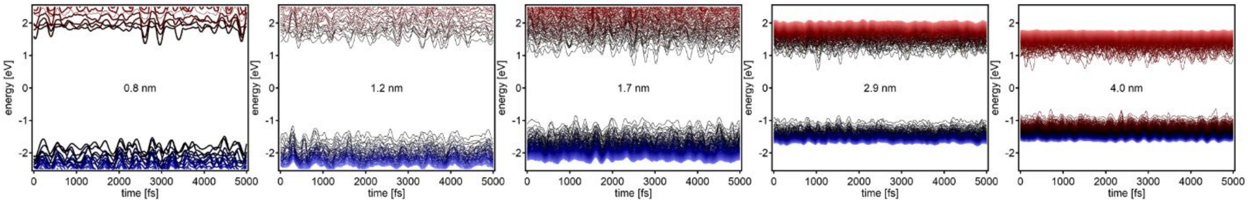

Figure S3 Conduction and valence orbital energies for $CsPbBr_3$ NCs from 0.8 to 4.0 nm during an MD trajectory of 5 ps.

Energy- and state-resolved electron cooling kinetics

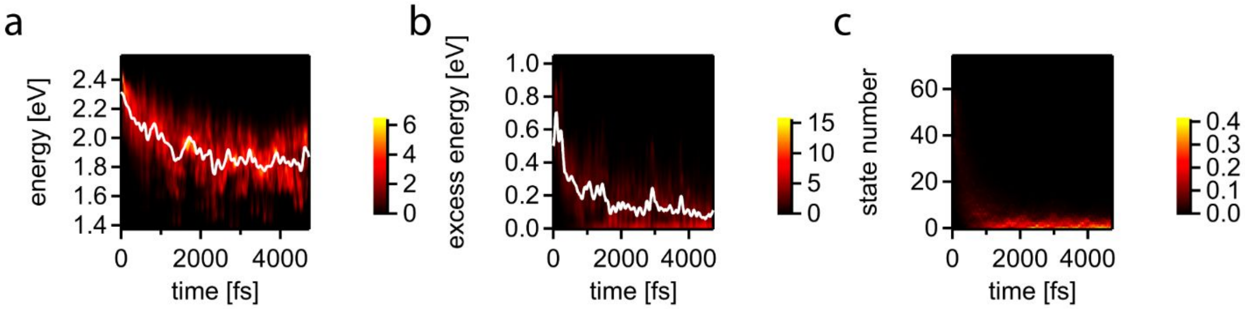

Figure S4 Electron cooling kinetics computed for a 4.0 nm $CsPbBr_3$ NC. (a) absolute energy. (b) excess energy with respect to the instantaneous lowest-energy conduction band state, *i.e.* the

CBM newly evaluated for each time point in the trajectory. (c) state-resolved electron cooling, where the conduction band state numbers are labeled with respect to the CBM, *i.e.* 0 = CBM, 1 = CBM + 1, *etc.*

Maxwell-Boltzmann fit to electron and hole populations of NAMD simulations

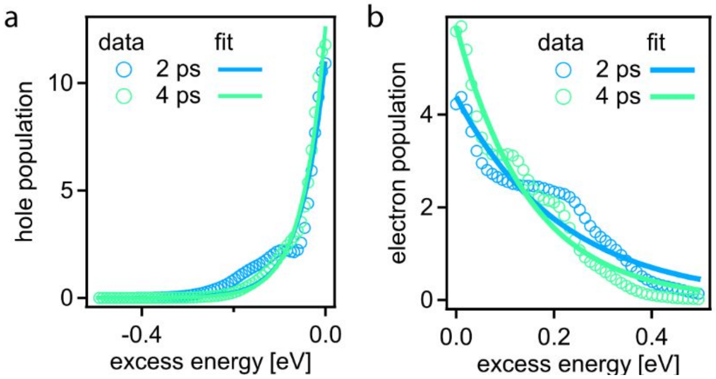

Figure S5 Electron (a) and hole (b) populations in 4.0 nm $CsPbBr_3$ NCs at 2 and 4 ps, depicted by blue and green open circles, respectively, and representing the data shown in Figure 3 of the main text. Fits assuming a Maxwell-Boltzmann distribution (representing the tail of a Fermi-Dirac distribution far above the Fermi level) are shown as solid lines.

Table S1. Electron and hole excess energies defined as the 1/e energies from the Maxwell-Boltzmann fits in Figure SX

	Electron	Hole
**2 ps**	0.218 ± 0.017 eV	-0.053 ± 0.004 eV
**4 ps**	0.150 ± 0.009 eV	-0.049 ± 0.002 eV

Fit of the decay of the average excess energy in NAMD simulations

We fit the average excess energy of electrons and holes (white lines in Figure 3b of the main text) with the decay model proposed by Prezhdo et al.,[14] extended by a second exponential component: after an initial Gaussian decay due to the "quantum Zeno effect", *i.e.* slow intraband transitions due to decoherence, the subsequent decay is assumed to proceed biexponentially, according to $\Delta E_{avg}(t) = a_g exp\left(-\frac{t^2}{t_g^2}\right) + a_{fast} exp\left(-\frac{t}{t_{fast}}\right) + a_{slow}\, exp\left(-\frac{t}{t_{slow}}\right)$, where $t_{g,fast,slow}$ and $a_{g,fast,slow}$ are the time constants and corresponding weights of the Gaussian, rapid exponential, and slow exponential decay component, respectively. We quantify the initial cooling stage comprised of the Gaussian and rapid exponential component *via* the sum of the weighted contributions, *i.e.* $t_{cool} = \left(\frac{a_g}{a_g+a_{fast}}\right) t_g + (\frac{a_{fast}}{a_g+a_{fast}}) t_{fast}$. For 4 nm NCs, this initial cooling time constant amounts to $t_{cool,e} = 0.69$ ps for the electron and $t_{cool,h} = 0.75$ ps for the hole, and slightly increases for both carriers in smaller NCs.

Fit of the NAMD kinetics with a static six-level cascade model

To rationalize the electron and hole cooling data obtained from the NAMD simulations shown in Figure 3a-d of the main text, we fit the kinetic traces in Figure 3d with a cascade model comprised of six static levels between 0 and 0.5 eV excess energy, arbitrarily spaced by 0.1 eV, see Figure 3e. We note that such a model is intended to enhance the conceptual clarity at the expense of a drastically simplified electronic structure. For example, the static model clearly neglects the large energy fluctuations of the electronic structure (see Figure 3a) and the level

spacing of 0.1 eV is a multiple of the real level spacing. Considering that for the considered 4.0 nm $CsPbBr_3$ NC, the six assumed levels in our model represent about 50 electron states and about 70 hole states, each transition between adjacent model levels represent on average about 8 and 12 transitions between adjacent “real” electron and hole levels, respectively.

Despite its drastic simplification of the electronic structure, the cascade model successfully fits the electron and hole cooling kinetics obtained from the NAMD simulations (see Figure 3d). It is important to note, though, that adequate fits were only obtained after inclusion of re-excitation options: as shown in Figure 3e, the cascade model allows both downward and upward transitions between adjacent levels, representing relaxation (*i.e.* ‘cooling’) and re-excitation (*i.e.* ‘heating’), respectively. The respective rates are $k_{down,i}$ and $k_{up,i}$, where $i = 1, 2, 3, 4, 5$ indicates the transition between levels i and $i - 1$ for downward transitions, and between levels $i - 1$ and i for upward transitions, respectively. As discussed in the main text, we interpret the necessity to include re-excitation by frequent state crossings of the real electronic structure (depicted in Figure 3a). In conclusion, favoring conceptual clarity over an exact description of the precise energetics, our cascade model was found useful in rationalizing the fast charge-carrier cooling at high excess energies and the surprisingly slow electron cooling close to the CBM.

Defining the excess energy via referencing to the CBM

In the Figures 3 and 4 of the main text as well as in Figure S4, we define the charge-carrier excess energy by referencing the carrier’s energy to the instantaneous lowest-energy state, at each time point in the trajectory. In principle, this represents an upper bound for the excess

energy, given the possibility that the thermal equilibrium state of the charge carrier is higher in energy than the thus defined CBM. To evaluate the extent to which thermal excitations may lead to overestimation of the electron excess energy, we also calculated the carrier's excess energy by referencing to an alternative definition of the CBM in which the CBM equals the average of all conduction band states, weighted by the Boltzmann-distribution at 300 K. Figure S6 shows that this alternative excess-energy referencing scheme results only in a minor (downward) correction of the excess energy by about 10 meV. Such minor energy correction in a quantum-confined NC is understandable, given that one expects the bulk value to equal $k_B T$ ~ 26 meV and 0 meV for very strongly confined NCs with intraband gaps >> $k_B T$. In summary, neither the time scale nor the excess energy of the persistent electrons reported in the main text are significantly affected by the specific type of defining the CBM. Per analogy, the same holds for the holes.

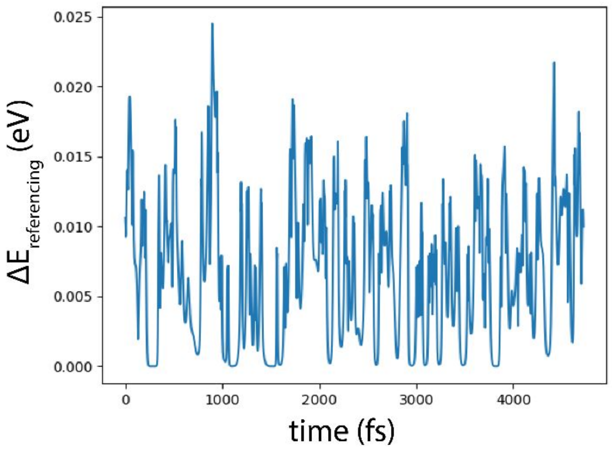

Figure S6. Overestimation of the excess energy obtained via referencing to the instantaneous lowest-energy state and the CBM defined as the average of Boltzmann-weighted energies.

Phonon DOS and phonon influence spectrum for electron and holes

The phonon DOS is obtained *via* a Fourier transformation of the autocorrelation of the nuclear positions in a 5 ps MD trajectory. The phonon-mediated decoherence loss between neighboring intraband states, expressed in Figure 5b as a ‘phonon influence spectrum’ for electrons and holes, is obtained *via* the auto-correlation function of intraband transition energies, see Neukirch et al. for details of this procedure.[11] Briefly, the phonon influence spectrum is calculated as $I(\omega) = \left|\frac{1}{\sqrt{2\pi}}\int_{-\infty}^{\infty} e^{-i\omega t} \cdot C(t)\right|^2$, where $C(t) = \langle\Delta E(t)\Delta E(0)\rangle/\langle\Delta E^2(0)\rangle$ is the normalized autocorrelation function for an intraband transition of energy E_{ij} between neighboring states $j = i+1$ and i, where $\Delta E = E_{ij} - \langle E_{ij}\rangle$ denotes the fluctuation with respect to the statistically averaged transition energy $\langle E_{ij}\rangle$.

REFERENCES

(1) De Roo, J.; Ibáñez, M.; Geiregat, P.; Nedelcu, G.; Walravens, W.; Maes, J.; Martins, J. C.; Van Driessche, I.; Kovalenko, M. V.; Hens, Z. Highly Dynamic Ligand Binding and Light Absorption Coefficient of Cesium Lead Bromide Perovskite Nanocrystals. ACS Nano 2016, 10 (2), 2071–2081. https://doi.org/10.1021/acsnano.5b06295.

(2) Maes, J.; Balcaen, L.; Drijvers, E.; Zhao, Q.; De Roo, J.; Vantomme, A.; Vanhaecke, F.; Geiregat, P.; Hens, Z. Light Absorption Coefficient of CsPbBr 3 Perovskite Nanocrystals. J. Phys. Chem. Lett. 2018, 9, 3093–3097. https://doi.org/10.1021/acs.jpclett.8b01065.

(3) Hens, Z.; Moreels, I. Light Absorption by Colloidal Semiconductor Quantum Dots. J. Mater. Chem. 2012, 22 (21), 10406. https://doi.org/10.1039/c2jm30760j.

(4) Chen, K.; Gallaher, J. K.; Barker, A. J.; Hodgkiss, J. M. Transient Grating Photoluminescence Spectroscopy: An Ultrafast Method of Gating Broadband Spectra. J. Phys. Chem. Lett. 2014, 5 (10), 1732–1737. https://doi.org/10.1021/jz5006362.

(5) Richter, J. M.; Chen, K.; Sadhanala, A.; Butkus, J.; Rivett, J. P. H.; Friend, R. H.; Monserrat, B.; Hodgkiss, J. M.; Deschler, F. Direct Bandgap Behavior in Rashba-Type Metal Halide Perovskites. Adv. Mater. 2018. https://doi.org/10.1002/adma.201803379.

(6) Li, M.; Goh, T. W.; Sum, T. C.; Bhaumik, S.; Kumar, M. S.; Yantara, N.; Grätzel, M.; Mhaisalkar, S.; Mathews, N. Slow Cooling and Highly Efficient Extraction of Hot Carriers in Colloidal Perovskite Nanocrystals. Nat. Commun. 2017, 8, 14350. https://doi.org/10.1038/ncomms14350.

(7) Bussi, G.; Donadio, D.; Parrinello, M. Canonical Sampling through Velocity Rescaling Polymorphic Transitions in Single Crystals: A New Molecular Dynamics Method Canonical Sampling through Velocity Rescaling. J. Chem. Phys. J. Chem. Phys. J. Appl. Phys. J. Chem. Phys. J. Chem. Phys. J. Chem. Phys. J. Chem. Phys. 2007. https://doi.org/10.1063/1.2408420.

(8) Zapata, F.; Ridder, L.; Hidding, J.; Jacob, C. R.; Infante, I.; Visscher, L. QMflows: A Tool Kit for Interoperable Parallel Workflows in Quantum Chemistry. J. Chem. Inf. Model. 2019. https://doi.org/10.1021/acs.jcim.9b00384.

(9) Akimov, A. V.; Prezhdo, O. V. The PYXAID Program for Non-Adiabatic Molecular Dynamics in Condensed Matter Systems. J. Chem. Theory Comput. 2013. https://doi.org/10.1021/ct400641n.

(10) Yazdani, N.; Bozyigit, D.; Vuttivorakulchai, K.; Luisier, M.; Infante, I.; Wood, V. Tuning Electron-Phonon Interactions in Nanocrystals through Surface Termination. Nano Lett. 2018. https://doi.org/10.1021/acs.nanolett.7b04729.

(11) Neukirch, A. J.; Hyeon-Deuk, K.; Prezhdo, O. V. Time-Domain Ab Initio Modeling of Excitation Dynamics in Quantum Dots. Coordination Chemistry Reviews. 2014. https://doi.org/10.1016/j.ccr.2013.08.035.

(12) Brehm, M.; Kirchner, B. TRAVIS - A Free Analyzer and Visualizer for Monte Carlo and Molecular Dynamics Trajectories. J. Chem. Inf. Model. 2011. https://doi.org/10.1021/ci200217w.

(13) Thomas, M.; Brehm, M.; Fligg, R.; Vöhringer, P.; Kirchner, B. Computing Vibrational Spectra from Ab Initio Molecular Dynamics. Phys. Chem. Chem. Phys. 2013. https://doi.org/10.1039/c3cp44302g.

(14) Kilina, S. V.; Neukirch, A. J.; Habenicht, B. F.; Kilin, D. S.; Prezhdo, O. V. Quantum Zeno Effect Rationalizes the Phonon Bottleneck in Semiconductor Quantum Dots. Phys. Rev. Lett. 2013. https://doi.org/10.1103/PhysRevLett.110.180404.
